# Durable Antibacterial Performance of Au–Ag–Cu Thin Films Prepared by Magnetron Sputtering: Real-World Applications

**DOI:** 10.3390/molecules30163348

**Published:** 2025-08-12

**Authors:** Agata Markowska-Szczupak, Rafał J. Wróbel, Anna Kiełbus-Rąpała, Beata Michalkiewicz

**Affiliations:** 1 Department of Chemical and Process Engineering, Faculty of Chemical Technology and Engineering, West Pomeranian University of Technology in Szczecin, Piastow Ave. 42, 71-065 Szczecin, Poland; agata.markowska@zut.edu.pl (A.M.-S.); anna.kielbus-rapala@zut.edu.pl (A.K.-R.); 2 Department of Catalytic and Sorbent Materials Engineering, Faculty of Chemical Technology and Engineering, West Pomeranian University of Technology in Szczecin, Piastow Ave. 42, 71-065 Szczecin, Poland; rafal.wrobel@zut.edu.pl

**Keywords:** magnetron-sputtering method, antibacterial, pen, copper, gold, silver

## Abstract

The growing prevalence of bacteria resistant to antibiotics and conventional disinfectants is a cause for concern and underscores the necessity of developing new strategies to prevent the transmission of microorganisms. To this end, nanocrystalline Cu, Au, and Ag nanoparticles were employed to fabricate various coatings using the sputtering technique. Then, the antibacterial activity of the coatings against Gram-negative *Escherichia coli* and Gram-positive *Staphylocococcus epidermidis* was investigated. The coating obtained by co-sputtering of Au, Ag, and Cu exhibited the most pronounced antibacterial properties. This coating was applied to disposable BIC ballpoint pens, which were subsequently used by clients in two public institutions. After three months of regular use, the antibacterial properties of the coatings were re-evaluated. It was confirmed that this coating led to a significant reduction (log5 CFU/mL) in the bacterial presence on the treated surface within 0.5 h. These results support further investigation into the underlying mechanism, which is likely based on the synergistic interaction of the employed noble metal nanoparticles.

## 1. Introduction

While the SARS-CoV-2 pandemic has largely faded from public discourse, antimicrobial resistance remains an ongoing global health challenge. Notably, the most recent antibiotic introduced into clinical practice (bedaquiline, BDQ) dates back to the early 2010s [1,2]. Unfortunately, bacterial resistance to BDQ in isolates from tuberculosis patients was first reported as early as 2015 [3]. For that reason, in many countries, the treatment of diseases caused by drug-resistant Mycobacterium tuberculosis is not known [4]. With increased use of antibiotics across the world, there is growing concern about the rapid rise of antimicrobial resistance (AMR) and multidrug-resistant (MDR) strains. Examples include methicillin-resistant *Staphylococcus aureus* (MRSA), multidrug-resistant *Pseudomonas aeruginosa* (MDR-PA), and multidrug-resistant *Acinetobacter baumannii* (MDR-AB) [5]. The main dangers of this phenomenon are incurable bacterial infections. A recent report suggests that drug-resistant bacteria will kill approximately 10 million people annually by 2050 [6]. The following factors are likely to contribute to the rise of antibiotic-resistant pathogens: (i) increasing use of a broad spectrum of antibiotics to treat infections, (ii) use of antibiotics in agriculture, (iii) an aging population, and (iv) the growing use of antiseptics and disinfectants in the home, in particular during the COVID-19 pandemic in 2019 [7]. Disinfectants are chemical agents that are especially used on hard surfaces and in water to reduce the concentration of microorganisms to a safe level and help to prevent the transmission of diseases. As with antibiotics, the overuse of disinfectants may lead to bacterial resistance. Decreased susceptibility to disinfectants may be induced by mutations or amplification of endogenous chromosomal genes by acquisition of resistance determinants on chromosomal genetic elements such as plasmids, transposons, etc. Another bacterial adaptation strategy includes inactivation or neutralization of the disinfectant as well as biofilm formation [8,9].

The best hope for developing a new generation of anti-infective agents is to discover new methods of treatment. A wide range of available nanoparticles appears capable of circumventing established bacterial drug-resistance mechanisms [10]. Moreover, the application of nanomaterials offers a promising solution to reduce bactericidal agent use for cleaning medical devices, surfaces, and critical areas in hospitals as well as in the household. According to Rutala et al. [11], the widespread use of antibacterial products in homes can contribute to the development of antibiotic-resistant Gram-negative bacteria (including the common *Escherichia coli*) and Gram-positive bacteria (e.g., *Staphylococcus aureus*).

In our previous work, entitled “Fabrication of antibacterial metal surfaces using magnetron-sputtering method”, we described the use of a magnetron-sputtering method for the preparation of thin bimetallic films consisting of silver, copper, and gold and their antibacterial properties [12]. The purpose of this work is to present the antimicrobial activity of a new coating and its possible mechanisms of action, as well as the application potential when applied to the surfaces of ballpoint pens.

## 2. Results

### 2.1. Thin-Film Characteristics

**Figure 1:**
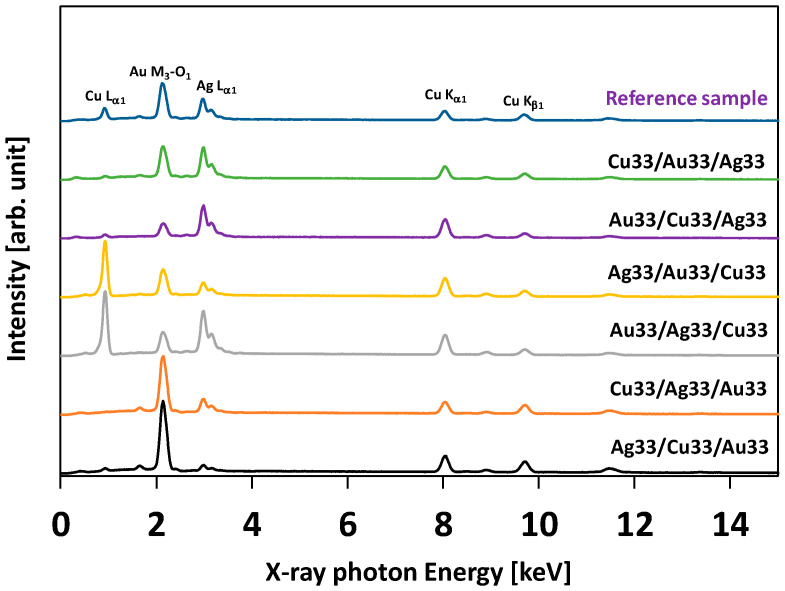
EDS spectra of prepared systems and of the reference sample.

Figure 1 presents the EDS spectra of the prepared systems obtained using an accelerating electron voltage of 25 kV. The main signals, corresponding to copper, gold, and silver, are indicated. The notation Au33/Ag33/Cu33 refers to a sandwich-like sequence of metal layers—starting from the top, copper, followed by silver in the middle and gold at the bottom—deposited on the substrate.

This sequence denotes that signals below copper will be screened and attenuated. Therefore, the Cu Lα_1_ signals are strongest for the Ag33/Au33/Cu33 and Au33/Ag33/Cu33 samples. Conversely, the Cu Lα_1_ signals are weakest for the Cu33/Au33/Ag33 and Cu33/Ag33/Au33 samples. This attenuation effect can be observed for the signals of all three metals. In order to prove this concept, a special sample of a mixture/alloy of three metals was prepared and tagged as the reference sample. This sample did not consist of a sequence of metal layers but was a single layer of a mixture/alloy of metals. This is why the Cu Lα_1_, Au M3-O1, and Ag Lα_1_ signals in the reference sample are of middling intensity compared with other spectra. The data are consistent with a screening effect. The EDS software that delivered the quantitative results assumes the homogeneity of investigated systems. Due to the screening effect caused by the inhomogeneous sandwich-like structure of the prepared systems, the quantitative EDS results are not given. We did not perform EDS mapping to visualize the distribution of elements. This was omitted for two main reasons. First, the films were expected to be homogeneous due to the fabrication process; the sample was rotated while nanoparticles were gradually deposited over the surface, ensuring uniform coverage. Second, the spatial resolution of the EDS technique coupled with SEM is limited—approximately 1000 nm at best. Therefore, although some inhomogeneities might have existed at the nanometric scale, they were beyond the detection capabilities of the EDS/SEM system.

**Figure 2:**
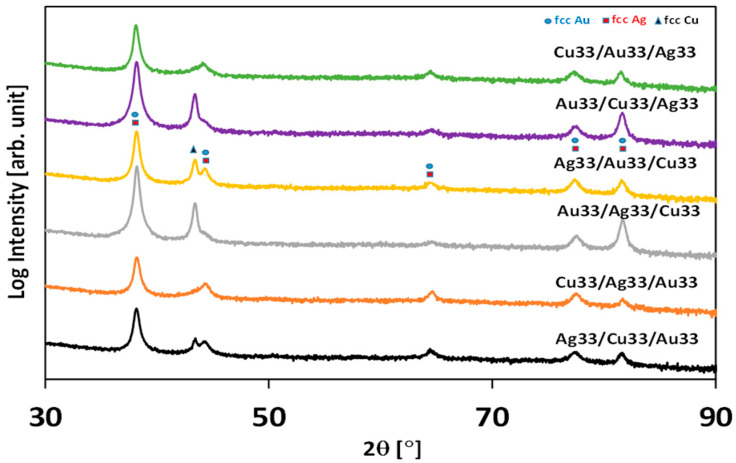
XRD spectra of prepared triple-metal-layer systems.

Figure 2 presents the XRD spectra of the prepared systems. All metals exhibit face-centered cubic (FCC) crystallographic systems. The diffraction peaks of gold and silver overlap each other. The exception is the reflex originating from copper above 40 degrees. A visible attenuation effect can be observed; i.e., the reflex originating from copper is the most pronounced for systems with copper topmost layers (Au33/Ag33/Cu33; Ag33/Au33/Cu33). Conversely, the copper reflex is mostly attenuated in samples where the copper layer is at the bottom (Cu33/Ag33/Au33; Cu33/Au33/Ag33). For systems where the copper layer is in the middle, the attenuation effect is stronger when the gold layer is on the top (Ag33/Cu33/Au33) compared to silver as a topmost layer (Au33/Cu33/Ag33). The explanation of this effect is the higher atomic number of gold than silver. The heavier elements attenuate stronger X-rays compared with lighter elements. Similar effects have been observed for double-layered systems [12].

**Figure 3:**
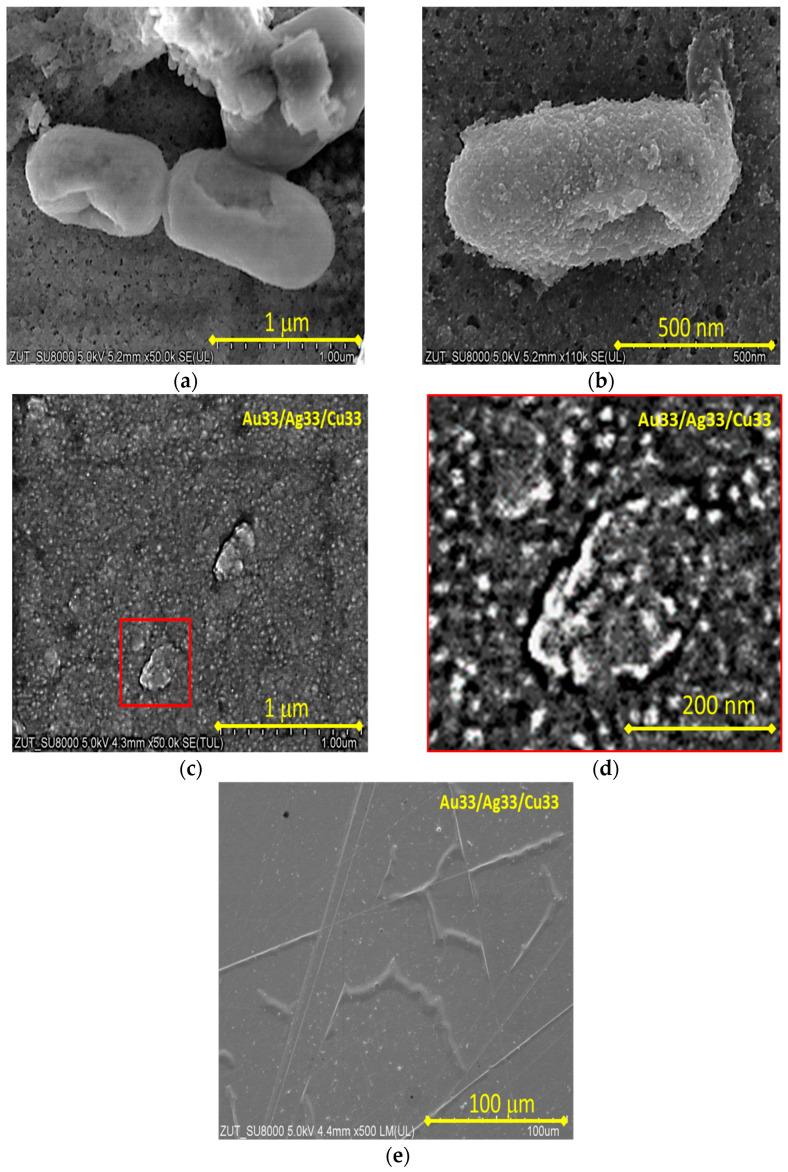
SEM images of prepared triple-metal-layer systems on an example of a Au33/Ag33/Cu33 sample: (**a**,**b**)—dead bacteria on the surface; (**c**,**d**)—grainy structure; and (**e**)—defects in the macroscale.

Figure 3 presents SEM images of the obtained samples. Figure 3a,b present the bacteria over the sample surface. The sizes of the individual bacteria are about 1 µm each. Figure 3c shows the surface of the Au33/Ag33/Cu33 sample at a magnification of 50,000×. The SEM images of all the samples were similar; therefore, the Au33/Ag33/Cu33 sample is presented as an example. An area marked in Figure 3c is further magnified and shown in Figure 3d. Clusters of metal with sizes of approximately 20 nm are visible. The grainy structure of the nanometric size may have resulted in higher specific surface areas of the obtained metallic films compared to coarse-grained metal materials. The higher specific surface area may result in higher antibacterial properties because such materials release metallic ions more rapidly in aqueous solutions. Figure 3e shows macroscopic defects, such as scratches and delamination of the metallic layer from the plastic substrate.

### 2.2. Antibacterial Tests

**Figure 4:**
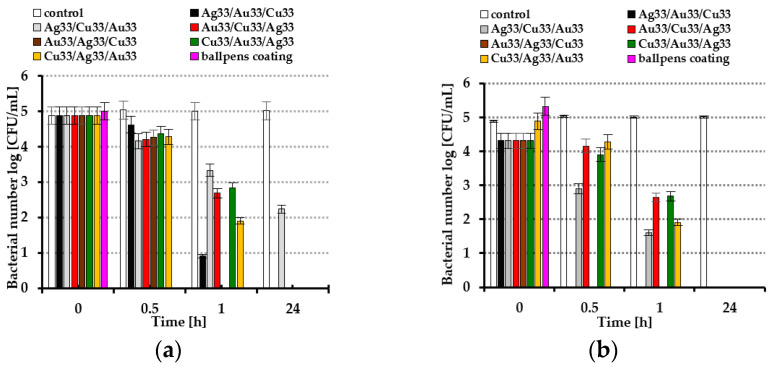
Bacterial number log [CFU/mL] of (**a**) *Escherichia coli* and (**b**) *Staphylococcus epidermidis*. The data are representative of the three independent experiments using triplicate samples, and means ± SD values are presented.

In Figure 4a,b, the antimicrobial properties of the tested magnetron-sputtered coatings are shown. This study was conducted according to the dynamic-contact ASTM E2149-20 standard test with our own modification. The layer obtained by simultaneous sputtering of Ag, Au, and Cu particles on the plastic surfaces of ballpoint pens (pink columns in Figure 4a,b) presented the best germicidal features against Gram-negative *E. coli* bacteria and Gram-positive *S. epidermidis* bacteria. After just half an hour, all bacteria were killed (Figure 4a,b). As expected, the other tested coatings obtained by subsequent application of metal layers on the plate presented varied antibacterial properties. The Au33/Ag33/Cu33 metallic surfaces rapidly (after 1 h) and efficiently killed *E. coli* and *S. epidermidis* bacteria (Figure 4a,b). The Ag33/Cu33/Au33 layers presented the worst disinfection properties against *E. coli* bacteria. Viable bacteria were still detected after 24 h on the glass surface (Figure 4a).

Considering the results presented above for coating ballpoint pens, the layer obtained by simultaneous sputtering of Ag, Au, and Cu particles was chosen. It was also decided to limit the duration of the following experiments to 1.5 h.

**Figure 5:**
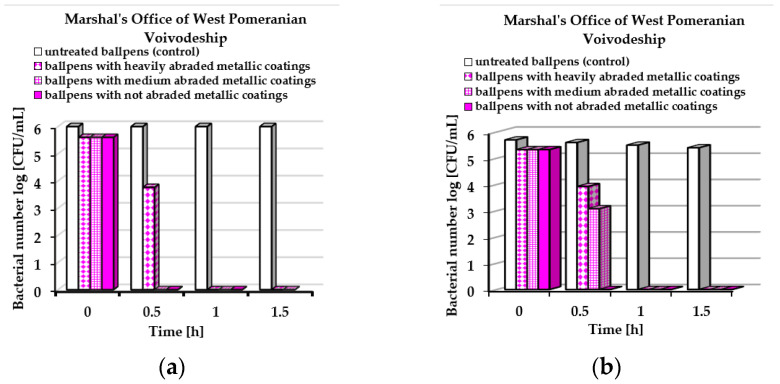
Average bacterial number log [CFU/mL] on the surface of ballpoint pens used in the Marshal’s Office of West Pomeranian Voivodeship detected by dynamic method: (**a**) *Escherichia coli* and (**b**) *Staphylococcus epidermidis*.

**Figure 6:**
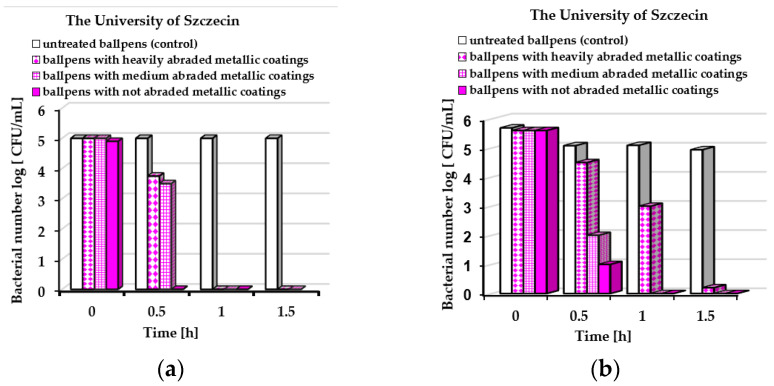
Average bacterial number log [CFU/mL] of ballpoint pens used at the University of Szczecin detected by dynamic method: (**a**) *Escherichia coli* and (**b**) *Staphylococcus epidermidis*.

In Figure 5 and Figure 6, the *E. coli* (Figure 5a or Figure 6a) and *S. epidermidis* (Figure 5b or Figure 6b) underwent reductions in the numbers of bacteria in contact with ballpoint pens covered by layers obtained by simultaneous sputtering of Ag, Au, and Cu particles on the plastic surfaces. The dynamic-contact ASTM E2149-20 standard test was applied. Fifty ballpoint pens were used by visitors of the Marshal’s Office of West Pomeranian Voivodeship and employees of the University of Szczecin within three months. The use of the ballpoint pens resulted in varying degrees of abrasion or wear, which is taken into account in Figure 5 and Figure 6.

Based on the obtained results, it can be stated that the degrees of wear of the ballpoint pens after use influenced their antibacterial performance. Moreover, varied results were obtained at both experimental sites. The best antibacterial properties were obtained for ballpoint pens with unabraded metallic coatings. After 1.0 h, all *E. coli* and *S. epidermidis* bacteria were killed regardless of the place where the experiment was conducted (Figure 5a,b or Figure 6a,b). The weakest antimicrobial effect was exhibited for ballpoint pens with heavily abraded metallic coatings. *S. epidermidis* bacteria were still detected after 1.5 h of exposure to ballpoint pens used at the University of Szczecin (Figure 6b).

**Figure 7:**
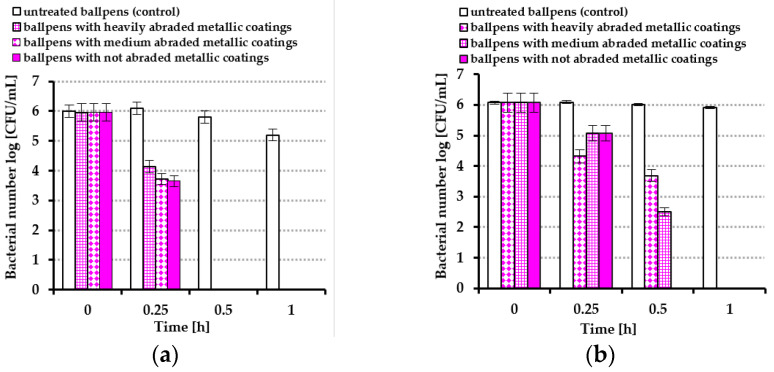
Average bacterial number log [CFU/mL] on the surfaces of ballpoint pens used at the University of Szczecin detected after nebulization onto the surface: (**a**) *Escherichia coli* and (**b**) *Staphylococcus epidermidis*.

Because this dynamic method enables the determination of bacterial reduction in a solution in which ballpoint pens are immersed in a buffer suitable for bacteria, it was decided to quantify the number of bacteria directly applied onto the ballpoint pens’ surfaces (this method is described in paragraph 4.8). For this purpose, a bacteria suspension was nebulized onto the surface of each ballpoint pen. In that experiment, only ballpoint pens used by employees of the University of Szczecin were used. The results are presented in Figure 7a,b.

As can be seen from Figure 7a, *E. coli* bacteria were killed on the surfaces of the ballpoint pens within only 0.5 h, regardless of the degree of coating damage on the pens. Following the same period of time, the Gram-positive bacterium *S. epidermidis* was still detected (Figure 7b).

**Figure 8:**
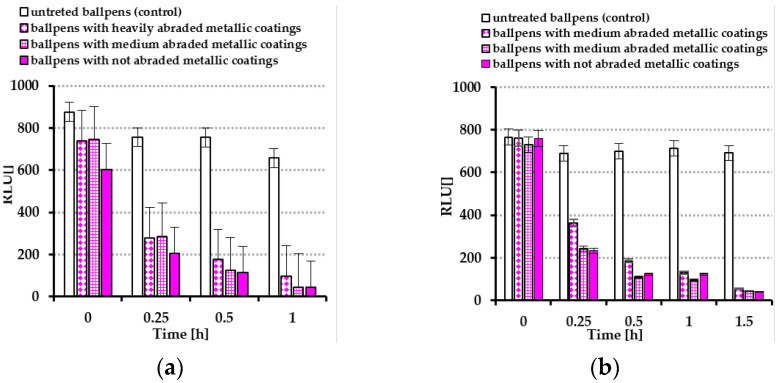
RLU measurements for ballpoint pens nebulized—(**a**) *Escherichia coli* and (**b**) *Staphylococcus epidermidis*—using ATP bioluminescence (Relative Light Units, RLUs).

To determine the presence of living bacteria, animal, and plant cells on the surfaces of ballpoint pens covered by a layer obtained by the simultaneous sputtering of Ag, Au, and Cu particles on each plastic surface, the bioluminescence method was used. Figure 8a shows the results for the ballpoint pens treated via nebulization with an *E. coli* suspension, whereas Figure 8b presents the results for *S. epidermidis.*

Initially high RLU values (exceeding 800) were detected, indicating that this directly correlated with the numbers of bacteria present on the ballpoint pen surfaces’ coatings. After only 0.25 h, the ballpoint pen surfaces were cleaned of *E. coli* and *S. epidermidis* bacteria to acceptable levels, defined as bioluminescence of < 200 RLU. For the ballpoint pens not coated with metallic nanolayers, the RLU values remained high even after 1.5 h (Figure 8a,b).

## 3. Discussion

Different magnetron-sputtering methods, such as radio frequency (RF) or high-power impulse magnetron sputtering (HiPIMS), are used in industrial applications to deposit coatings on large surfaces [13]. These techniques are associated with relatively low operational costs, but the obtained coatings display varying characteristics influenced by the deposition technique selected and the type of nanoparticles employed [14].

The effects of the various coatings containing noble metal nanoparticles such as gold, silver, and copper on microbial growth were tested. In addition, the suitability of the most effective coating was evaluated for use in coating ballpoint pens used by multiple users (employees and service recipients) in two public service institutions in Szczecin (the University of Szczecin and Marshal’s Office of West Pomeranian Voivodeship). To test the antibacterial activity of the varied obtained coatings in terms of viable colonies of *E. coli* and *S. epidermidis*, two colony-forming unit assays were performed as direct quantification methods. The magnetron-sputtering method was used to apply thin layers of metals on glass and the surfaces of widely available BIC ballpoint pens. As predicted based on our previous analysis of bimetallic coatings [12], trilayer coatings with copper as the outermost layer exhibited the most effective antibacterial properties. It was also confirmed that Gram-negative bacteria are less susceptible to inactivation upon contact with noble metal nanocoatings produced via magnetron sputtering than Gram-positive bacteria [12] (Figure 4a,b). Surprisingly, in our study, a coating obtained by simultaneous sputtering of Ag, Au, and Cu particles had a strong antibacterial effect directly on the surfaces of glass and ballpoint pens. It has been shown that on the glass plates after 0.5 h, all *E. coli* and *S. epidermidis* bacteria were killed. When the coating was applied to the plastic surfaces of the ballpoint pens, the bactericidal effect depended on the degree of coating wear during normal use as well as on exposure time. It was found that no viable bacteria were detected after 1 h. As shown by experiments conducted by Kaltschmidt et al. [15], glass coatings with transition metals—gold, ruthenium, and tantalum—deposited by the magnetron-sputtering method prevent bacterial biofilm formation. The growth of Gram-negative *Pseudomonas aeruginosa* isolated from household appliances was analyzed by scanning electron microscopy (SEM). The best antibacterial properties were obtained when the surface was covered with a 20 nm-thick tantalum coating, whereas ruthenium was less efficient. High-resolution SEM images depicted bacterial cell wall destruction and completely destroyed bacteria on the metal surfaces. In the present study, we obtained similar images of bacteria showing structural damage (Figure 3a,b). The formation of reactive oxygen species (ROS) was considered a potential mechanism [15]. It is generally believed that free radicals cause peroxidation of lipids and proteins and DNA degradation and disturb the internal ROS balance, leading to oxidative stress that induces toxicity of metal and metal oxide nanoparticles. Their disinfection potential relies on the interaction between microorganisms and ROS, such as hydroxyl radicals and superoxide ions, under exposure to light or magnetic fields [16]. Interaction with atmospheric oxygen and water on the surfaces of noble metal nanoparticles can also catalyze redox reactions, leading to the generation of reactive oxygen species (corrosion reactions) [17]. However, the effectiveness of ROS disinfection is influenced by many factors. During photocatalytic reactions, the effectiveness depends on the type of photocatalyst, medium in which the photocatalyst is suspended, surface characteristics of photocatalysts, organic matter content (acting as scavengers), and other environmental conditions, whereas metal corrosion reactions are highly material-specific and linked to both particle size and surface characteristics [16,17,18]. The formation of ROS is initiated by dissolved metal ions. In our previous study, we attributed the enhanced antimicrobial properties of copper-based outer layers to the oxidative dissolution of copper and subsequent release of Cu^2+^ ions from CuO. A synergistic effect of the coexistence of both metallic species was also indicated in our previous findings [12]. The results presented in this work support the validity of this hypothesis. When the coating obtained by simultaneous sputtering of Ag, Au, and Cu particles was applied to ballpoint pens, it was found that the degree of its wear, resulting from regular use, determined its antibacterial properties. Two experimental approaches were used to evaluate this: a dynamic method, in which pens were immersed in a buffer solution appropriate for the specific bacterial species, and a spray method, in which a bacterial suspension was sprayed onto the surfaces of dry ballpoint pens. In both approaches, the bacteria were suspended in an aqueous environment. It is beyond doubt that this could have triggered corrosion reactions of metallic coatings and ROS production. The nanocoating developed in the present studies is expected to demonstrate enhanced antibacterial performance in humid conditions. The use of ballpoint pens while writing may have contributed to increased hand sweating and mild moisture accumulation. It should be noted that hand moisture can vary. At rest, in a dry environment, healthy human hands are usually dry, although they may feel slightly moist to the touch due to the skin’s natural secretions [19].

**Figure 9:**
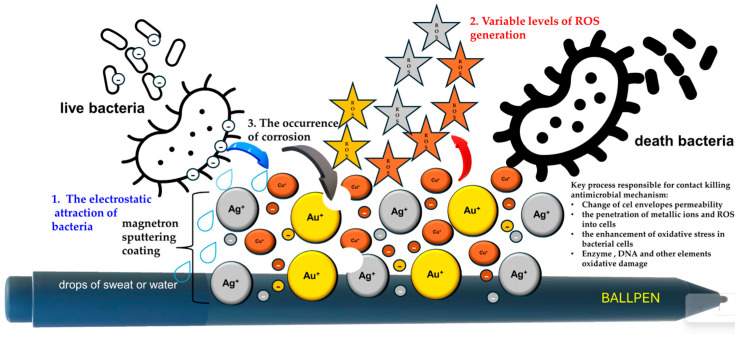
Proposed mechanism of antibacterial action of coating obtained by simultaneous sputtering of Ag, Au, and Cu particles.

Furthermore, under aqueous conditions, the zeta potential of nanoparticles can significantly affect the antimicrobial activity of obtained coatings against studied bacteria. As has been widely reported, Gram-negative bacteria exhibit a significantly lower (more negative) zeta potential compared to Gram-positive bacteria [20]. According to Saed et al. [21], the zeta potential of gold nanoparticles (NPs) is of great importance for antibacterial properties. It determines the stability of NPs and their electrostatic interactions with each other as well as with other entities (including bacteria). Nanoparticles with higher positive zeta potential values exhibit greater (stronger attraction of bacterial cells) antibacterial activity against negatively charged bacterial cell membranes [21]. During regular use of the ballpoint pens for writing, progressive abrasion of the applied coating was observed. This wear occurred gradually and did not result in visible residue on users’ hands, indicating good adherence of the coating material. After approximately three months of intensive use, ballpoint pens classified as ”heavily abraded” (<50% uncovered surfaces) were considered unsuitable for further use, typically due to complete ink depletion or ink leakage (as commonly observed in disposable pens, Figure 9). Despite this advanced stage of wear, antimicrobial testing revealed that the coatings maintained a significant level of antibacterial activity (Figure 5, Figure 6 and Figure 7). This suggests that the biocidal components remained on the surface or were effectively re-exposed during abrasion, underscoring the durability and long-term effectiveness of the coating under real-life conditions. This was verified through bioluminescence testing of the coatings. This method relies on a chemical reaction where adenosine triphosphate (ATP) reacts with luciferase, producing light, which is measured by a luminometer (Kikkoman Lumitester Smart ATP, used in our study). ATP is a sensitive indicator molecule for the presence of biological residues due to its ubiquity and presence in all living cells (from microbes to animal and plant cells), while RLUs (Relative Light Units) are the units for chemiluminescent reactions that generate light output linearly to the amount of ATP present in living cells or biological residues [22,23]. Bioluminescence tests cannot replace microbial tests. There are no quantitative tests for bacterial counts, and they cannot be used as precision assays for detection of microbial contamination. However, bioluminescence tests are fast, direct tests of surface cleaning efficiency. According to many authors, bioluminescent ATP-metry is used to determine the contamination levels of the surfaces of equipment at food factories and in some biotechnological processes [22,24]. The high rate of luminescent reactions (high RLU values) allows quick detection of hazardous production areas and gives information about possible contamination of surfaces or other objects. The results obtained in these investigations are in excellent agreement with the data collected from both previous microbial tests. The results indicate that the nanometallic coatings applied on the surfaces of ballpoint pens lead to rapid reduction in bacterial viability. A significant decrease in RLUs was observed at as early as 0.25 h. After 1.5 h, the RLU level dropped below 50 units, indicating that the ATP detection limit had been reached.

Coatings obtained by simultaneous sputtering of Ag, Au, and Cu particles can be successfully used to cover both everyday items and objects that are frequently touched by people. Obtaining abradable surfaces designed to wear away under specific conditions might be useful for medical and surgical instruments. These coatings comprise three distinct types of nanoparticles, each exhibiting unique physicochemical properties and documented antibacterial activity. The notably rapid bacterial inactivation observed on these surfaces suggests that the co-sputtering process may result in the formation of a structurally and functionally complex nanosystem. Further physicochemical and biological characterization is required to fully understand the interactions within this system and their contribution to the observed antimicrobial performance. The enhanced antibacterial efficacy of Ti-Cu thin films obtained by the co-sputtering method was also demonstrated by Mahmoudi-Qashqay et al. [25]. The influence of the deposition method and the sequence of the layer arrangement was also described by Vibornijs et al. [26]. Those authors demonstrated that multilayer coatings of ZnO/Cu/ZnO exhibit lower antibacterial activity against *E. coli* and *S. aureus* compared to a metal dopant (Cu) incorporated into the ZnO layer. Notably, the ZnO/Cu/ZnO coating exhibited a marked enhancement in ROS generation, likely due to the synergistic interaction between ZnO and Cu [26]. In this study, the same metal nanoparticles as previously used were employed for the preparation of coatings. Their crystallite sizes were approximately Cu—5 nm, Ag—50 nm, and Au—56 nm [12]. As demonstrated by Kessler et al. [17], nanoparticle size is one of the main factors influencing ROS generation. A schematic representation of our proposed antibacterial mechanism is shown in Figure 9.

Despite the growing body of research on antibacterial coatings, many studies remain confined to laboratory conditions. This limitation may stem from the absence of standardized methodologies, particularly when it comes to testing coated objects of larger dimensions or irregular geometry. To address this gap and to assess coating durability under practical application conditions, we selected ballpoint pens as model objects in the present study. Future studies should focus on elucidating the synergistic mechanisms of action of Cu, Ag, and Au nanoparticles against bacteria. This is difficult due to the fact that all redox reactions responsible for bacterial inactivation take place either on the surfaces of nanoparticles or in their immediate surroundings, with intermediates represented by adsorbed, chemisorbed, and desorbed species.

## 4. Materials and Methods

### 4.1. Magnetron Sputtering

Thin metallic coatings were deposited using the magnetron sputtering technique in a Q150T coater (Quorum Technologies, Sacramento, CA, USA), which was equipped with a turbomolecular pump. The deposition was carried out under the following conditions: ambient temperature (20 °C), base pressure of 1.0 × 10^−2^ mbar, argon flow rate of 50 cm^3^/min, and sputtering power of 40 W in direct-current (DC) mode. A quartz crystal microbalance sensor, placed at the same height as the sample substrates, was used to monitor film thickness with a precision of 0.1 nm. Substrates were mounted on a rotating platform located 10 cm beneath the metal target. High-purity metal targets (99.99%) of silver, gold, and copper, each with a 57 mm diameter, served as the source materials.

The deposition took place at a working pressure of 2 mbar in an argon plasma environment. Nanometric layers (100 nm total thickness) were deposited over the surfaces of plastic ballpoint pens. During deposition, a constant ion current of 100 mA was maintained. The resulting sample series consisted of six three-layer films: Ag33/Cu33/Au33; Cu33/Ag33/Au33; Au33/Ag33/Cu33; Ag33/Au33/Cu33; Au33/Cu33/Ag33; and Cu33/Au33/Ag33. For the samples, the notation indicates the order of layer deposition—for instance, Ag33/Cu33/Au33 corresponds to a stack structured as substrate/33 nm Ag/33 nm Cu/33 nm Au, with Au being the topmost layer. A reference sample was also prepared by co-deposition of silver, gold, and copper. The round target was composed of three equal segments, each occupying one-third of the area and made of a pure metal. The resulting sample was labeled the reference sample.

### 4.2. Scanning Electron Microscopy (SEM) Coupled with Energy-Dispersive Elemental Analysis (EDX)

High-resolution imaging of the thin metal films was conducted using a field emission scanning electron microscope (SU8020; Hitachi Ltd., Tokyo, Japan, 2012) that utilizes a cold field emission electron source. The instrument was magnetically shielded to ensure image stability at ultra-high magnifications, reaching up to 200,000× magnification. Specimens with metal nanofilms were affixed to the sample stage using conductive carbon tape. To mitigate surface charging effects, the conductive tape was also used to provide electrical grounding to the sample. SEM imaging was performed at an accelerating voltage of 5 kV.

For elemental characterization, X-ray emission analysis was carried out using an Energy-Dispersive Spectroscopy (EDS) detector (UltraDry; Thermo Scientific, Pittsburgh, PA, USA). During EDS analysis, the samples were exposed to a primary electron beam with an energy of 25 keV to induce characteristic X-ray generation.

### 4.3. X-Ray Diffraction (XRD)

Structural analysis of the thin metal films was carried out using a versatile X-ray diffractometer (Empyrean; PANalytical, Malvern, UK) fitted with a Cu-anode X-ray source (Cu Kα radiation, λ = 0.15406 nm). Measurements were performed over a 2θ range from 30° to 90°, with an increment of 0.026°. Phase identification and data interpretation were conducted using X’Pert HighScore Version 3.0e (3.0.5) software in combination with the PDF-4+ 2020 database from the International Centre for Diffraction Data (ICDD). All analyzed metallic layers revealed a face-centered cubic (FCC) crystal structure. The corresponding ICDD reference patterns used for Cu, Ag, and Au were 04-001-3178, 04-003-5319, and 01-089-3697, respectively.

### 4.4. The Ballpoint Pen Experiment

**Figure 10:**
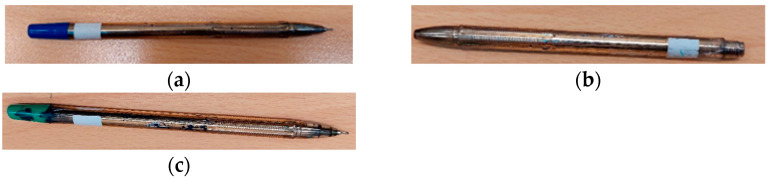
Appearances of sample ballpoint pens: (**a**) ballpoint pen with unabraded metallic coating (<50% uncovered surfaces); (**b**) ballpoint pen with medium abraded metallic coating (approx. 50% uncovered surfaces); and (**c**) ballpoint pen with heavily abraded metallic coating (approx. 80–90% uncovered surfaces).

The ballpoint pens covered by films obtained by magnetron sputtering were distributed in the gatehouses of two public utility institutions: the University of Szczecin (50 pieces) and Marshal’s Office of West Pomeranian Voivodeship (50 pieces). The ballpoint pens were used to record clients’ entries and exits. This experiment was conducted during three months (March–June 2020). The pens were then collected and tested according to the method described in paragraph 4.8. Based on normal wear and tear, the ballpoint pens were divided into three groups: (I) ballpoint pens with heavily abraded metallic coatings (approximately 80–90% uncovered surfaces); (II) ballpoint pens with medium-abraded metallic coatings (approximately 50% uncovered surfaces); and (III) ballpoint pens with unabraded metallic coatings (<50% uncovered surfaces). Examples of the ballpoint pens are presented in Figure 10.

### 4.5. In Vitro Susceptibility Test

For the microbial test, we chose the same bacteria as in our previous studies [10]. These were Gram-negative *Escherichia coli,* strain K12 ATCC 25922 (*E. coli*), and Gram-positive *Staphylococcus epidermidis*, ATCC 49461 (*S. epidermidis*). We pre-cultivated the *E. coli* in Enrichment Broth (BIOCORP Sp. z.o.o., Warszawa, Poland) and *S. epidermidis* in Brain Heart Infusion Broth (BHI) (BIOCORP Sp. z.o.o., Poland) for 24 h at 37 °C. Then, the overnight cultures of bacteria were diluted with appropriate buffers (0.85% NaCl sterile saline buffer for *E. coli* and TBS buffer for *S. epidermidis*) until the final concentration of bacteria was in the range of approx. 1.5–3.0 × 10^6^ CFU/mL (working solution). All buffers and media were prepared according to the manufacturer’s recommendations. For the test, we used BIC Cristal—inexpensive, widely available ballpoint pens with transparent barrels and refills (Société Bic, Clichy, France). The antimicrobial tests were conducted according to the dynamic-contact ASTM E2149-20 standard test in our own modification [27]. The modification involved the use of a different *E. coli* strain, as well as the use of a model strain of Gram-positive bacteria (*S. epidermidis*), the collection of extra samples after 0.5 and 1.5 h, and a different method of presenting the results. In the first approach, the pen barrels (without refills) covered with various magnetron coatings and the untreated pen barrels (negative control) were sterilized under a UV-C lamp Alpina BIO190, Konin, Poland for 15 min. Five treated and untreated pen barrels were placed into a sterile buffer in 800 mL Simax glass bottles. Then, 200 ± 0.5 mL of the working dilution of prepared bacterial inoculum was added to the bottles. The magnetic stir bars were set in bottles, which were placed on a magnetic stirrer in an incubator at a temperature of 37 °C. Then, 0.5 mL was taken as a sample after 0.5, 1, and 1.5 h. The bacterial concentrations at the “0” time and during the experiment were measured by serial dilutions and standard plate-count techniques in triplicate. Plate-count agar (PCA) (BIOCORP Sp. z.o.o., Poland) was used for the *E. coli* and Brain Heart Infusion Agar (BHI) (BIOCORP Sp. z.o.o., Poland) for the *S. epidermidis*. After 24 h of incubation at 37 ± 2 °C for 24 h, the visible bacteria colonies were counted. The results were presented as log of colony-forming units per milliliter (CFU/mL).

In the second approach, a 5.5 mL bacterial working solution containing *E. coli* or *S*. *epidermidis* bacteria (approx. 1.0–1.1 × 10^6^ CFU/mL) was spread on pens by nebulization. This experiment was conducted in a specially constructed aerosol deposition chamber. After 0, 0.25, 0.5, and 1 h, the ballpoint pens were rinsed with 10 mL of 0.85% NaCl sterile saline buffer. Then 0.5 mL was diluted and placed, according to the procedure described above. Only ballpoint pens (50 pieces) used by visitors of the University of Szczecin were tested.

Finally, ATP bioluminescence detection of all organic residues and microorganisms on the surfaces of the pen barrels was performed using the Kikkoman Lumitester Smart ATP Hygiene Monitoring System, model 61234 (Kikkoman Biochemifa Company, Noda, Japan). Measurements were carried out using the LuciPac A3 Surface swab Kikkoman Biochemifa Company, Noda, Japan after 0.25, 0.5, and 1 h. The absence of any interference of the microorganisms with the ATP bioluminescence reaction was assumed based on the indications of the bioluminometer supplier. Samples were measured in triplicate with a registration time of 5 s. The average results of the three ATP measurements were expressed as Relative Light Units (RLUs).

## 5. Conclusions

It has been found that the complex relationship between surface morphology, structure, and surface chemistry profoundly influences the interaction between sputter-deposited coatings and bacterial cells. Understanding these parameters is essential for optimizing coating design to maximize antimicrobial efficacy. Significant reductions in model Gram-positive (*S. epidermidis*) and Gram-negative (*E. coli*) bacteria, >5 log CFU/mL, were observed after just 0.5 h on the coating obtained by simultaneous sputtering of Ag, Au, and Cu. It was confirmed that this coating can be effectively applied to small everyday objects such as ballpoint pens, buttons, door handles, smartphone cases, and similar everyday items, demonstrating its practical potential. Moreover, the coating maintained full antibacterial efficacy for a duration of three months under typical usage conditions, highlighting its potential for long-term antimicrobial protection.

In conclusion, our findings may contribute to the development of effective antibacterial coatings as a promising complement or alternative to conventional disinfectants, particularly in everyday applications where frequent manual disinfection is impractical or undesirable.

## 6. Patents

The following patent are associated with the results presented in this manuscript: Patent Number: PL435460; Title: A component made of metal, plastic, glass, or fabric and a method for producing antimicrobial barriers; Inventors: R. Wróbel, et al.; Filing Date: 2020-09-28; Status: Pending and Patent Number: PL438138; Title: A plastic component and a method for producing an antimicrobial barrier; Inventors: R. Wróbel, et al.; Filing Date: 2021-06-14; Status: Pending.

## Data Availability

Data is contained within the article. Further inquiries can be directed to the corresponding author (A.M.-S.) and co-author (R.J.W.).

## Abbreviations

The following abbreviations are used in this manuscript:

AMR	Antimicrobial resistance
ATCC	American Type Culture Collection
ATP	Adenosine triphosphate
BDQ	Bedaquiline
BHI	Brain Heart Infusion Agar
BIC	Inexpensive, disposable ballpoint pens mass-produced and sold by Société Bic of Clichy, Hauts-de-Seine, France
CFU	Colony-forming unit
EDS	Energy-Dispersive Spectroscopy
HiPIMS	Impulse magnetron sputtering
MDR	Multidrug-resistant bacteria
MDR-AB	Multidrug-resistant *Acinetobacter baumannii*
MDR-PA	Multidrug-resistant *Pseudomonas aeruginosa*
MDR-SA	Methicillin-resistant *Staphylococcus aureus*
NPs	Nanoparticles
PCA	Plate-count agar
RF	Radio frequency sputtering
ROS	Reactive oxygen species
RLU	Relative Light Unit
SEM	Scanning electron microscopy
TBS	Tris-buffered saline
XRD	X-ray diffraction
